# The Persian Version of the Quick Mild Cognitive Impairment Screen (Q*mci*-Pr): Psychometric Properties among Middle-Aged and Older Iranian Adults

**DOI:** 10.3390/ijerph18168582

**Published:** 2021-08-14

**Authors:** Mohammad Rezaei, Behnam Shariati, David William Molloy, Rónán O’Caoimh, Vahid Rashedi

**Affiliations:** 1 Autism Spectrum Disorders Research Center, Hamadan University of Medical Sciences, Hamadan 6517838687, Iran; m_r_st@yahoo.com; 2 Mental Health Research Center, Department of Psychiatry, School of Medicine, Iran University of Medical Sciences, Tehran 1449614535, Iran; behnamshariatimd@gmail.com; 3 Department of Geriatric Medicine, Mercy University Hospital, T12 WE28 Cork, Ireland; w.molloy@ucc.ie (D.W.M.); rocaoimh@hotmail.com (R.O.); 4 School of Behavioral Sciences and Mental Health (Tehran Institute of Psychiatry), Iran University of Medical Sciences, Tehran 1445613111, Iran

**Keywords:** cognitive decline, screening, psychometrics, older adults

## Abstract

Brief cognitive screening instruments are used to identify patients presenting with cognitive symptoms that warrant further assessment. This study aimed to evaluate the reliability and validity of the Persian version of the Quick Mild Cognitive Impairment (Q*mci*-Pr) among middle-aged and older Iranian adults. Consecutive patients aged ≥55 years and caregivers attending with them as normal controls (NCs) were recruited from geriatric outpatient clinics and a hospital in Tehran, Iran. All patients completed the Q*mci*-Pr before completing an independent detailed neuropsychological assessment and staging using the Clinical Dementia Rating (CDR) Scale. NCs underwent the same assessment. In all, 92 participants with a median age of 70 years (±13) were available. Of these, 20 participants were NCs, 24 had subjective memory complaints (SMC), 24 had mild cognitive impairment (MCI), and 24 had Alzheimer’s disease (AD). The Q*mci*-Pr had good accuracy in differentiating SMC and NC from MCI (area under the curve (AUC): 0.80 (0.69–0.91)) and in identifying cognitive impairment (MCI and mild AD) (AUC: 0.87 (0.80–0.95)) with a sensitivity of 88% and specificity of 80%, at an optimal cut-off of <53/100. The Q*mci*-Pr is an accurate short cognitive screening impairment for separating NC and patients with SMC from MCI and identifying cognitive impairment. Further research with larger samples and comparison with other widely used instruments such as the Montreal Cognitive Assessment is needed. Given its established brevity, the Q*mci*-Pr is a useful screen for Iranian adults across the spectrum of cognitive decline.

## 1. Introduction

Neurocognitive disorders are a broad class of impairments in cognition, usually associated with aging [1,2]. Neurocognitive disorders are classified as minor, including mild cognitive impairment (MCI), and when functional impairment is established, as major (i.e., dementia) [3]. In dementia, cognitive impairment hinders independence in everyday functioning. People with MCI remain autonomous [4], although they may already have subtle deficiencies when performing complex activities [5]. Data show that approximately half of the patients diagnosed with MCI will transition to dementia within a short number of years [6].

The treatment and diagnosis of cognitive impairment have become an important public health issue in both developed regions and, increasingly, in developing low- and middle-income countries [7]. The prevalence of neurocognitive disorders, including both MCI [8] and dementia [9], is rising worldwide. Despite this, a higher proportion of cases are underdiagnosed, with more than 80% of reported cases of MCI going unrecognized in primary care [10]. While there is limited evidence supporting routine screening in clinical practice [11], as MCI is a transitional state between normal cognition and dementia, if identified early, it presents an opportunity for interventions, including population-level public health approaches to slow progression towards dementia [12]. Moreover, from a clinical management perspective, treatment options for people with MCI differ from those with dementia [13]. It is essential to differentiate between normal cognition, MCI, and dementia because treatment choices differ. Patients with MCI, for example, do not benefit from the initiation of cholinesterase inhibitors [14]. In order to correctly identify those with early cognitive impairment (MCI or mild dementia), reliable, valid, simple, and short cognitive screens are required.

Several validated cognitive screening instruments are used to differentiate normal cognition from MCI and dementia [15], including the Mini-Mental State Examination (MMSE) [16] and the Montreal Cognitive Assessment (MoCA) [17]. The Quick Mild Cognitive Impairment (Q*mci*) screen is a new, brief, and reliable tool. It was designed to sensitively recognize the specific differences between MCI and normal controls (NCs) [18], and has a quick administration time of approximately five minutes [19]. It is recognized and communicated in English in countries, including Canada [18] and Australia [20,21], as well as multiple other languages, including Turkish [22], Chinese [23], and Dutch [24]. The Q*mci* screen has six subtests that cover five cognitive domains: orientation, working memory, semantic memory (verbal fluency), visuospatial (clock drawing), and two tests of episodic memory (delayed recall and logical memory) [25]. 

At present, with respect to clinical practice in Iran, particularly in busy hospital clinics, there is insufficient evidence to show that suitably accurate, sensitive, and specific short cognitive screening instruments are able to differentiate MCI from normal aging and dementia. Given this concern, this study aimed to evaluate the reliability, concurrent, and convergent validity of the Persian (Farsi) version of the Quick Mild Cognitive Impairment (Q*mci*-Pr) among middle-aged and older Iranians.

## 2. Materials and Methods

We conducted a cross-sectional observational study at geriatric clinics at the Rasoul Akram hospital (affiliated with the Iran University of Medical Sciences) to evaluate the psychometric properties of the Q*mci*-Pr screen. Consecutive patients attending geriatric outpatient clinics complaining of memory loss were recruited between January 2019 and November 2019. Community-dwelling patients aged ≥55 years were eligible. The sample included normal controls (NCs), recruited from participants’ caregivers and controlled with standard testing and without subjective symptoms. Patients were diagnosed with subjective memory complaints (SMC) if they reported problems with or changes in memory but had normal cognitive testing. Those with MCI and patients with Alzheimer’s disease (AD) were diagnosed according to the criteria outlined in the *Diagnostic and Statistical Manual of Mental Disorders, 5th Edition* (*DSM-5*) [3]. Individuals younger than 55 years of age, as well as those diagnosed with other dementia subtypes, including frontotemporal dementia, Parkinson’s disease, or Lewy body dementia, which presents infrequently, typically with excessive functional deficits and different MCI syndromes, were excluded from the study. Where possible, all participants provided written informed consent; when deemed unable to provide this, assent was sought. This study was approved by the Ethics Committee of the Hamadan University of Medical Sciences (Ref No.: IR.UMSHA.REC.1396.461). Ethical approval was obtained in advance, and the study was conducted according to the Declaration of Helsinki.

The participant’s diagnostic category was determined by a trained and experienced psychiatrist who differentiated diagnostic groups independent of (impartial to) the Q*mci*-Pr screen results. The Q*mci*-Pr was scored in advance by trained raters working in the clinic. Sociodemographic data such as age, gender, marital status, education level, and bilingualism were collected. All participants, including NC and patients referred to the clinic, underwent comprehensive neuropsychological assessments and were staged using the Persian version of the Clinical Dementia Rating (CDR) scale [26]. The Geriatric Depression Scale (GDS) was used to screen for depression.

The Q*mci*-Pr has six subtests: orientation (country, year, month, day, and date; 10 points in total); a five-item word registration (5 points); a clock drawing test (15 points); a test of delayed recall of the items registered (20 points); a categorical verbal fluency test (20 points); and finally, a logical memory test, which evaluates the immediate verbal recall of a short story (30 points). The subtests can be administered and scored in five minutes or less for a total score of 100 points, with higher scores indicating better cognition. The English-language version has a cut-off score of <62/100 for cognitive impairment (MCI or dementia) and <50/100 for dementia [25]. Alternative versions are used to minimize learning effects. The Q*mci*-Pr was adapted using a forward-backward translation method. The CDR total score is used to characterize six cognitive and functional performance domains applicable to AD and related dementias: memory, orientation, judgment and problem solving, community affairs, home and hobbies, and personal care. Lotfi et al. showed that the CDR scale is a sensitive and precise test for assessing and staging cognition in the Iranian geriatric population [26]. The CDR scale was used to assess concurrent validity. 

The GDS-15 is a 15-item self-report scale that is used to identify depression among older adults and is commonly used as a routine part of a comprehensive geriatric assessment. In a study by Malakouti et al., the Persian version of GDS showed excellent properties as a screening instrument for major depression among older adults in Iran [27]. Here, it was used to support a clinical diagnosis of depression and to measure convergent validity.

Data were analyzed using SPSS version 22.0 (IBM Corp., Armonk, NY, USA). Bivariate correlations assessed the relationship between instruments, and the differences in test scores and demographic variables between groups were tested using one-way analysis of variance (ANOVA) and chi-squared tests (χ^2^). Internal consistency was measured using Cronbach’s alpha. Accuracy was obtained from the area under the curve (AUC) using receiver operating characteristic (ROC) curve analysis. AUC values between 0.60 and 0.69 were considered “poor”, those between 0.70 and 0.79 were considered “fair”, those between 0.80 and 0.89 were considered “good”, and those >0.90 as excellent. Cut-off scores were obtained using Youden’s Index. A *p*-value <0.05 was regarded as statistically significant.

## 3. Results

A total of 92 middle-aged and older adults agreed to participate and were included in this study. The mean age of the total sample was 69.76 ± 9.09 years, the majority of which were male (62%). The participants included 20 NCs, 24 with SMC, 24 with MCI, and 24 with AD. The mean ages of the NC, SMC, MCI, and AD groups were 64.4 ± 7.3 years, 68 ± 9.8 years, 72 ± 7.4 years, and 74 ± 8.9 years, respectively. The demographic and clinical characteristics of the total sample and the four groups are summarized in Table 1. Those with AD were older than the other diagnostic groups (*p* = 0.001). There were, however, no statistically significant differences in gender, marital status, education level, and bilingualism between the four diagnostic groups (*p* > 0.05). Lower cognitive performance on the Q*mci*-Pr and CDR scales was observed in the AD group (Table 1).

There was a significant correlation between Q*mci*-Pr scores and age (*r* = –0.328, *p* = 0.001), GDS scores (*r* = –0.213, *p* = 0.041), and CDR scores (*r* = –0.710, *p* < 0.001). The Q*mci*-Pr was significantly and negatively correlated with CDR for SMC and MCI (Table 2). Cronbach’s alpha for the Q*mci*-Pr was 0.81, which indicated a high degree of internal consistency and homogeneity between items. ROC curve analysis showed that the Q*mci*-Pr had similar accuracy in differentiating SMC and NC from MCI, with an AUC of 0.80, 95% confidence intervals (CI): 0.69–0.91, and good accuracy in identifying cognitive impairment (MCI and AD) with an AUC of 0.87 (95% CI: 0.80–0.95), a sensitivity of 88%, and specificity of 80%, at an optimal cut-off of <53/100 (Table 3).

## 4. Discussion

The present study was designed to determine the reliability and validity of the Persian (Farsi) version of the Q*mci* screen. The results suggest that the Q*mci*-Pr, a new, quick, and practical short cognitive screening instrument for middle-aged and older Iranian adults in clinical practice, is reliable and can be used to screen for cognitive impairment and triage those requiring further evaluation. The Q*mci*-Pr has high sensitivity and specificity and sufficient diagnostic accuracy in differentiating NC and SMC from MCI and AD. The Q*mci*-Pr also had moderate accuracy in separating MCI from AD among patients presenting with cognitive symptoms. The results showed that the Q*mci*-Pr strongly correlated with the CDR scale and a significant negative correlation with GDS scores, indicating convergent validity. In the SMC group, the GDS and Q*mci*-Pr did correlate significantly, suggesting that their low scores may reflect low mood as well as impaired cognition. This supports studies suggesting that those with SMC may have both depression and cognitive impairment. This result is also consistent with literature that reports that depression and cognitive scores are negatively correlated [28]. The present study also showed a high degree of internal consistency and homogeneity between items of the Q*mci*-Pr, confirming that the instrument had good construct validity among middle-aged and older Iranians and confirming the results of other studies [23,29]. 

The diagnostic accuracy of the Q*mci*-Pr is also similar to other studies examining the Q*mci* screen in different languages and countries, where it has been shown to have greater accuracy than the standardized MMSE and MoCA [30]. This study suggests that the optimal cut-off for the Q*mci*-Pr is <38 for AD and <53 for cognitive impairment (either MCI and SMC). The cut-off for cognitive impairment found in this sample varies from other language versions of the Q*mci,* including countries such as Ireland (<61), Canada (<62), and Japan (<60) [31,32,33]. However, it was more similar to some language versions, including the Turkish (<48), Italian (<49), and Greek (<51) versions [22,29,34]. 

There are several possible reasons for this variance. Differences in the sample size may have accounted for this, as smaller samples produce less generalizable results. For example, the sample size has varied from 100 patients in the Turkish study [22] to 92 participants in this study, to as high as 3387 in a pooled analysis from Canada [32]. Further, lower levels of education and higher illiteracy levels in some countries likely explain the lower cut-off scores (Varan et al., 2020). In this study, relatively high illiteracy levels were consistent with a lower mean number of years of education among Iranians, particularly older Iranians, albeit few data are available for these generations [35].

This study also has several limitations. The small sample size and the small number of outpatient clinics for data collection (limited to one city) may reduce the findings’ generalizability. The small number of participants in individual diagnostic groups may also have introduced bias. Furthermore, the difference between the proportion and mean value between groups influences the power of Q*mci*-Pr to differentiate between diagnostic groups and cannot be excluded, thereby limiting the interpretation of findings. That being said, there were no differences in gender, marital status, education level, and levels of bilingualism between those in each category. Further, the results, including cut-off scores, are similar to the findings of other studies examining older patients with lower levels of education. The diagnosis of MCI and AD was based on clinical symptoms, and biomarkers were not obtained, which may have extended this group’s heterogeneity and led to some bias. Similarly, a detailed neuropsychological assessment was not performed. However, the widely accepted CDR scale was used, and currently, there is no gold standard criterion for MCI. Given so, a wide variety of approaches to diagnosing MCI have been used in different studies [36]. Furthermore, significant differences in the GDS between groups could have caused confounding. Finally, this study did not compare the Q*mci*-Pr to other short cognitive screens, limiting the findings’ interpretability. Given these limitations, we suggest that further research with larger samples and comparison with other widely used instruments such as the Montreal Cognitive Assessment is now needed.

## 5. Conclusions

This study presents the first validation of the Persian version of the Q*mci* among middle-aged and older Iranians. It shows that the Q*mci*-Pr is accurate for screening cognitive impairment and separates NCs from those with SMC or MCI. Further research with larger samples and comparison with other widely-used instruments such as the MoCA is needed. Given its established brevity, the Q*mci*-Pr may be a useful screen across the spectrum of cognitive decline in an Iranian population. Given that early identification of cognitive impairment will become an increasingly important public health priority [37], the development of a brief but accurate and widely-acknowledged cognitive screening instrument [30] has important public health implications.

## Figures and Tables

**Table 1:** Demographic and clinical characteristics of the study sample.

Variable	Total		NC		SMC		MCI		AD		Test Value (df) of F Value	*p*-Value
	*N*/M	%/SD	*N*/M	%/SD	*N*/M	%/SD	*N*/M	%/SD	*N*/M	%/SD		
Gender											0.64 ^χ2^ (3)	0.887
Male	57	62	11	55	15	62.5	16	66.7	15	62.5		
Female	35	38	9	45	9	37.5	8	33.3	9	37.5		
Marital Status											3.72 ^χ2^ (6)	0.714
Single	2	2.2	0	0	1	4.2	0	0	1	4.2		
Married	72	78.2	18	90	17	70.8	19	79.2	18	75		
Divorced	18	19.6	2	10	6	25	5	20.8	5	20.8		
Education Level											14.63 ^χ2^ (17)	0.262
Illiterate	12	13	3	15	3	12.5	3	12.5	3	12.5		
Basic School	20	21.7	1	5	5	20.8	7	29.2	7	29.2		
Middle School	16	17.4	8	40	2	8.3	2	8.3	4	16.7		
High School	20	21.7	5	25	5	20.8	5	20.8	5	20.8		
Academic	24	26.2	3	15	9	37.5	7	29.2	5	20.8		
Bilingual											1.21 ^χ2^ (3)	0.750
Yes	67	72.8	7	65	5	79.2	6	75	7	70.8		
No	25	27.2	13	35	19	20.8	18	25	17	29.2		
Age	69.76	9.09	64.4	7.30	67.8	9.8	72	7.4	74	8.9	5.75	0.001
GDS	6.13	4.11	4.25	2.59	8.58	4.83	5.33	3.48	6.04	3.98	5.16 ^F^ (3)	0.002
Q*mci*-Pr (Total)	47.67	21.32	70	10.13	54	17.31	47	11.16	24	14.82	43.04 ^F^ (3)	<0.001
Orientation	7.86	2.83	9.80	0.69	9.08	1.74	8.21	2.22	4.67	2.79		
Registration	4.07	1.17	4.85	0.48	4.17	1.00	4.25	0.84	3.12	1.42		
Clock Drawing	9.92	6.15	14.40	0.99	11.54	5.52	10.04	5.69	4.46	5.90		
Delayed Recall	8.64	6.07	13.65	3.77	9.96	5.09	8.96	4.38	2.83	5.46		
Verbal Fluency	7.39	4.43	11.48	3.33	8.71	4.17	6.69	3.24	3.35	2.63		
Logical Memory	9.80	6.52	15.80	5.68	10.42	5.62	8.67	4.07	5.33	6.28		
CDR (Total)	3.17	3.85	0.35	0.36	1.97	2.27	2.45	2.16	7.45	4.55	26.81 ^F^ (3)	<0.001
Memory	0.64	0.80	0.05	0.15	0.33	0.50	0.56	0.47	1.54	0.88		
Orientation	0.37	0.64	00	00	0.10	0.25	0.22	0.36	1.08	0.82		
Community Affairs	0.71	0.89	0.10	0.20	0.54	0.76	0.58	0.77	1.54	0.90		
Judgment & Problem Solving	0.40	0.65	0.07	0.18	0.10	0.25	0.41	0.38	0.97	0.96		
Home & Hobbies	0.70	0.89	0.10	0.20	0.72	0.87	0.45	0.48	1.41	1.10		
Personal Care	0.33	0.67	0.02	0.11	0.16	0.24	0.20	0.44	0.89	1.05		

NC: Normal Control; SMC: Subjective Memory Complaints; MCI: Mild Cognitive Impairment; AD: Alzheimer’s disease; N: Number; M: Mean; SD: Standard Deviation; df: degree of freedom; GDS: Geriatric Depression Scale; Q*mci*-Pr: Quick Mild Cognitive Impairment screen—Persian (Farsi) version; CDR: Clinical Dementia Rating scale.

**Table 2:** Correlations between Q*mci*-Pr screen scores and age, GDS and CDR scale scores in the four diagnostic groups NC, SMC, MCI, and AD.

Group	Variable	Q*mci*-Pr	
		*r*	*p*-Value
NC	Age	–0.445	0.054
	GDS	–0.080	0.738
	CDR	–0.267	0.255
SMC	Age	–0.137	0.522
	GDS	–0.626	0.001
	CDR	–0.774	<0.001
MCI	Age	–0.067	0.755
	GDS	–0.318	0.130
	CDR	–0.701	<0.001
AD	Age	0.238	0.264
	GDS	0.173	0.418
	CDR	–0.207	0.332

Q*mci*-Pr: Quick Mild Cognitive Impairment screen—Persian (Farsi) version; NC: Normal Control; SMC: Subjective Memory Complaints; MCI: Mild Cognitive Impairment; AD: Alzheimer’s disease; GDS: Geriatric Depression Scale; CDR: Clinical Dementia Rating scale.

**Table 3:** Diagnostic properties of the Persian version of the Quick Mild Cognitive Impairment screen (Q*mci*-Pr), including optimal cut-off scores.

Diagnostic Classification	AUC (95% CI)	Sensitivity (%)	Specificity (%)	Youden’s Index	Cut-off
Cognitive Impairment (MCI and AD) vs. No MCI	0.87 (0.80–0.95)	88%	80%	0.68	<53
MCI vs. AD	0.88 (0.78–0.99)	88%	88%	0.76	<38
MCI vs. NC and SMC	0.80 (0.69–0.91)	79%	80%	0.59	<53
AD vs. No AD (NC, SMC and MCI)	0.92 (0.86–0.92)	88%	90%	0.78	<38

AUC: Area under the operating characteristic curve; CI: Confidence Intervals; MCI: Mild Cognitive Impairment; AD: Alzheimer’s disease; NC: Normal Control; SMC: Subjective Memory Complaints.

## Data Availability

The data sets generated and analyzed during the current study are available from the corresponding author upon reasonable request.
